# Functional neuroimaging in subjective cognitive decline: current status and a research path forward

**DOI:** 10.1186/s13195-020-00591-9

**Published:** 2020-03-09

**Authors:** Raymond P. Viviano, Jessica S. Damoiseaux

**Affiliations:** 1 Department of Psychology, Wayne State University, 5057 Woodward Ave. 7th Floor Suite 7908, Detroit, MI 48201 USA; 2 Institute of Gerontology, Wayne State University, 87 E. Ferry St., Detroit, MI 48202 USA

**Keywords:** Subjective cognitive decline, Functional neuroimaging, Connectivity

## Abstract

Subjective cognitive decline is a putative precursor to dementia marked by perceived worsening of cognitive function without overt performance issues on neuropsychological assessment. Although healthy older adults with subjective cognitive decline may function normally, perceived worsening may indicate incipient dementia and predict future deterioration. Therefore, the experience of decline represents a possible entry point for clinical intervention. However, intervention requires a physical manifestation of neuroabnormality to both corroborate incipient dementia and to target clinically. While some individuals with subjective cognitive decline may harbor pathophysiology for specific neurodegenerative disorders, many do not display clear indicators. Thus, disorder-agnostic brain measures could be useful to track the trajectory of decline, and functional neuroimaging in particular may be sensitive to detect incipient dementia and have the ability to track disease-related change when the underlying disease etiology remains unclear. Therefore, in this review, we discuss functional neuroimaging studies of subjective cognitive decline and possible reconciliations to inconsistent findings. We conclude by proposing a functional model where noisy signal propagation and inefficient signal processing across whole-brain networks may lead to the subjective experience of decline and discuss future research directions guided by this model.

## Introduction

Subjective cognitive decline (SCD) is a putative precursor to dementia marked by professed worsening of cognitive functioning, in any domain, without explicit performance issues on neuropsychological assessment. Precise definition and labeling of the concept have evolved over the past decades, with an international working group recently coming to a consensus to help spur data consistency [1]. Briefly, “subjective” denotes personal experience and can be orthogonal to objective function. For example, an individual with anosognosia may not notice worsening cognitive ability but perform poorly on assessment, while an individual with perception of decline may perform within a normal range [2]. Although not a concurrent indicator of objective decline, evidence suggests SCD can predict future cognitive deterioration and may demarcate incipient dementia [3–7]. The term “cognitive” implies that perceived worsening may apply to any domain, not just memory function. This ensures that research efforts capture trajectories from SCD to dementia when initial symptoms are unrelated to memory. Many earlier definitions focused on perceived *memory* impairment [8]; however, *cognitive* is a more beneficial label than *memory* as individuals may misreport problems in other cognitive domains as memory problems. Finally, *decline* reflects a progressive experience of cognitive worsening rather than an acutely caused stable impairment. On an individual basis, decline may relate to healthy or pathological aging; regardless, the consensus label has shifted to *decline* from *impairment* to reflect a progressive nature—possibly due to underlying neurodegeneration.

The memory component of SCD has been the dominant focal point of prior research, and early data collection efforts emphasized memory deficit by determining SCD through a single question or a set of questions about perceived memory [8]. In addition, earlier definitions placed SCD as a stage directly before mild cognitive impairment on the healthy aging to Alzheimer’s disease (AD) continuum [9]. However, SCD has multiple etiologies and potential outcomes [1]; therefore, classification as a symptom or risk factor of incipient dementia may be more appropriate. Nevertheless, many reports discuss SCD within an AD framework. According to the modern research definition by the National Institute on Aging and the Alzheimer’s Association [10], *Alzheimer’s disease* is a designation for individuals who exhibit pathological amyloid-β and tau deposits, determined either through positron emission tomography or cerebrospinal fluid evaluation. Although discussion of SCD biases towards a precursor to AD, and SCD associates with future dementia development [1, 3], associations between SCD and amyloid-β and tau burden are mixed [11–18]; thus, an Alzheimer’s-centric approach could misguide interpretation of SCD data. Although AD may be the most common dementia etiology [19], and individuals with SCD may receive an AD diagnosis over time, SCD possibly serves more accurately as a branching point between cognitively intact aging and a myriad of potential outcomes, including various dementias but also, commonly, recovery to a subjectively unimpaired state. Indeed, for many, SCD may be a temporary event.

As there is no one-to-one mapping between SCD and preclinical AD, amyloid-β and tau burden evaluation alone are not the optimal strategies to characterize SCD. Furthermore, prior analyses and models propose that functional brain network communication may break down prior to amyloid-β accumulation [20, 21], suggesting that functional brain alterations are sensitive metrics for early change. Therefore, measures that are non-specific to disease outcome may be more appropriate to evaluate SCD than disease-specific indicators and could identify patterns of brain structure or function that discriminate between individuals who remain cognitively unimpaired from individuals who develop dementia at a later time. Furthermore, functional neuroimaging may be a viable method for tracking change in SCD as evidenced by previous longitudinal work showing change related to aging and neurodegenerative disease [22–24]. Therefore, here, we review the functional neuroimaging literature in SCD, propose a functional model of SCD where differences in signal processing across whole-brain networks may lead to the experience of cognitive decline, and suggest future research efforts to test the model. This model refers to individuals who experience SCD more likely due to nascent neurodegeneration than other causes and are at greater risk of developing dementia rather than individuals who exhibit cognitive maintenance or for whom SCD is a temporary event.

## Search and review methodology

We searched the PubMed and Web of Science Core databases for articles evaluating functional characteristics of the brain in SCD. Title, keyword, and abstract search terms included either subjective cognitive decline, subjective executive decline, subjective memory impairment, subjective cognitive impairment, subjective cognitive concerns, or subjective memory concerns. We then narrowed the results of the pooled broad searches with functional, connectivity, activity, magnetic resonance (fMRI), infrared spectroscopy, electroencephalography (EEG), and magnetoencephalography (MEG) as additional terms. After sifting through titles and abstracts, we identified 38 relevant articles. Table 1 provides an overview of the methods and sample characteristics for these prior analyses; it also provides more comprehensive overviews of specific results than we address in the body of the text. We excluded articles involving an intervention (e.g., diet or resveratrol), where SCD was a continuous variable measured within a patient population (e.g., subjective decline *in* Parkinson’s disease), where SCD was unrelated to aging (e.g., cancer, fatigue, multiple sclerosis), where there was no control group without cognitive concerns or no continuous measure of SCD, and when the methods did not include functional imaging.

**Table 1:** Results from various functional neuroimaging analyses in subjective cognitive decline

Authors	Year	Citation	Mod	Task/Rest	Methodology	Operationalization	Participants	Results	Pattern
Alexander et al.	2006	[25]	EEG	Eyes open and closed resting. Visual working memory task	Average power spectra over 4 s periods during resting state. Measure of phase synchrony during task.	Participants with SCD recruited from general population with complaint of memory problems increasing over time and informant confirmation. Controls age and gender matched.	79 SCD, 79 control	Greater α band power, increased frontal θ power, and increased spatiotemporal wave activity during working memory in SCD. Greater α power and wave activity related to lower verbal memory performance and reaction time, and greater reverse digit span performance.	Increase
Rodda et al.	2009	[26]	fMRI	Verbal episodic memory task	Voxelwise task contrasts. Group comparison with ANOVA. Cluster correction.	Participants with SCD had persistent memory concerns and were recruited from a memory clinic. Controls were recruited from a prior study and were similar to SCD in age and education.	10 SCD, 10 control	Greater left prefrontal cortex activation in SCD during encoding. No differences in recognition rate.	Increase
Maestu et al.	2011	[27]	MEG	Sternberg’s letter-probe task	MEG source reconstruction. Nonparametric permutation testing to identify spatio-temporal clusters that distinguish the groups.	Participants with SCD recruited from memory clinic with memory deterioration confirmed by informant. Participants scored below 9 on the Geriatric Depression Scale and greater than 13 on the Memory Failures of Everyday test. Healthy elderly control participants recruited from university educational courses.	12 SCD, 18 control	Greater task-related activation within 200–900 ms after stimulus onset in SCD in parietal, temporal, occipital, motor/premotor, and dorsal prefrontal regions.	Increase
Rodda et al.	2011	[28]	fMRI	Divided attention task with visual letters and auditory numbers	Voxelwise task contrasts. Group comparison with ANOVA. Cluster correction.	Participants with SCD had persistent perceived decline from prior memory performance and were recruited from a memory clinic. Controls were recruited as part of a prior study.	11 SCD, 10 control	Greater thalamus, caudate, posterior cingulate, hippocampus, and parahippocampus task-related activation in SCD. No differences on performance.	Increase
Dumas et al.	2013	[29]	fMRI	N-back working memory task	Voxelwise analysis. Group comparisons with random effects ANOVA (group by working memory load). Cluster-level correction.	Healthy, older, post-menopausal women recruited from general population. Participants identified as SCD if they endorsed > 20% of the items of the Cognitive Complaint Index and as control otherwise.	12 SCD, 11 control	Greater working memory task-related activity in precuneus, midfrontal, and cingulate gyri in SCD.	Increase
Hafkemeijer et al.	2013	[30]	fMRI	Eyes closed resting state	Independent components analysis and dual regression.	Participants with SCD recruited from memory outpatient clinic.	25 SCD, 29 control	Greater DMN and medial temporal connectivity in SCD.	Increase
Dillen et al.	2016	[31]	fMRI	Eyes open resting state	Functional seed-based correlation analysis.	Participants with SCD recruited from outpatient memory clinic. Healthy controls recruited from general population through advertisements. Participants with SCD had memory complaint scores > 24 and normal cognitive test results.	27 SCD, 25 control	Greater functional connectivity between retrosplenial cortex and ventromedial prefrontal cortex in SCD.	Increase
Sun et al.	2016	[32]	fMRI	Eyes closed resting state	Voxelwise amplitude of low-frequency fluctuation analysis. Group differences evaluated with voxelwise general linear model analysis.	Participants with SCD recruited from a memory clinic. Participants with SCD reported persistent decline in memory corroborated by an informant. Healthy controls recruited from the general population and did not harbor cognitive concerns.	25 SCD, 61 control	Greater low-frequency signal amplitude in superior temporal, cerebellar, occipital, and inferior parietal cortex in SCD. Greater low-frequency amplitude associated with poor verbal learning within the SCD group.	Increase
Cespon et al.	2018	[33]	EEG	Simon task	Evaluation of P300 latency and amplitude during executive control processes.	Participants recruited from general population and split into low-SCD and high-SCD groups based on scores of a memory complaints questionnaire.	18 low-SCD, 16 high-SCD	Greater medial prefrontal negativity during task associated with greater degree of subjective cognitive decline.	Increase
Lazarou et al.	2018	[34]	EEG	Administration of pictures of facial affect during EEG	N170 event-related potential evaluation.	Participants recruited from a memory clinic. SCD status based on SCD international working group suggestions.	14 SCD, 12 control	Greater N170 (negative) amplitude in response to faces expressing fear.	Increase
Verfaillie et al.	2018	[35]	fMRI	Resting state	Parcellation-based functional connectivity analysis. Linear regression evaluated strength of association between SCD and connectivity strength.	Healthy older adults recruited from general population if they had a family history of Alzheimer’s disease. Participants subsequently classified as SCD if they responded “Yes” to the question “Do you think your memory is becoming worse?”	68 SCD, 56 control	Greater connectivity between posterior DMN and medial temporal regions, in SCD.	Increase
Kawagoe et al.	2019	[36]	fMRI	Eyes closed resting state	Principle component multivariate pattern analysis for functional connectivity.	Participants recruited from the general population. Subjective memory score measured as a continuous variable.	155 participants with SCD as a continuous variable	Greater connectivity between lingual gyrus and cuneus, lingual gyrus and precuneus, superior parietal lobe and postcentral gyrus, and between cuneus and many occipital and association areas in SCD.	Increase
Bajo et al.	2012	[37]	MEG	Sternberg’s letter-probe task.	Functional connectivity patterns, via synchronization likelihood, evaluated for task hits.	Participants with SCD recruited from memory clinic with memory deterioration confirmed by informant. Participants scored below 9 on the Geriatric Depression Scale and greater than 13 on the Memory Failures of Everyday test. Healthy elderly control participants recruited from university educational courses.	12 SCD, 25 control	Diffuse lower synchronization between electrode pairs in α and β bands in SCD.	Decrease
Wang et al.	2013	[38]	fMRI	Eyes closed resting state	Independent components analysis and dual regression.	Participants recruited through advertisements and through referrals from medical centers. Group classification then based on results of neuropsychological assessment, self-, and informant-report. All participants classified by clinical consensus panel.	23 SCD, 16 control	Lower connectivity between right hippocampus and the DMN in SCD.	Decrease
Yasuno et al.	2015	[18]	fMRI	Eyes closed resting state	Region-of-interest based Pearson’s correlation functional connectivity analysis.	Participants with and without SCD recruited from hospital psychiatry unit. SCD classification based on Reisberg criteria.	23 SCD, 30 control	Lower functional connectivity between retrosplenial cortex and dorsomedial prefrontal cortex, and between retrosplenial and anterior cingulate cortex in SCD. No differences between groups in amyloidopathy.	Decrease
Lopez-Sanz et al.	2016	[39]	MEG	Eyes closed resting state	MEG source reconstruction and spectral analysis. Groups differences assessed with ANCOVA.	Participants recruited from a hospital neurology department, a center for the prevention of cognitive impairment, and a senior center. Cognitive concerns self-reported and SCD status determined by multidisciplinary panel.	39 SCD, 41 control	Diffuse lower relative α power in SCD. No differences in α peak frequency slowing.	Decrease
Contreras et al.	2017	[40]	fMRI	Eyes closed resting state	Independent components analysis resting-state network analysis.	Participants with SCD had cognitive concerns but tested normally on cognitive tests. Controls had no significant cognitive concerns.	16 SCD, 13 control	Greater functional connectivity within all resting-state networks negatively associated with the cognitive complaint index.	Decrease
Dillen et al.	2017	[41]	fMRI	Resting state	Region-of-interest based temporal network modeling.	Participants with SCD recruited from outpatient memory clinic. Healthy controls recruited from general population through advertisements. Participants with SCD had memory complaint scores > 24 and normal cognitive test results.	28 SCD, 25 control	Decreased functional connectivity between hippocampus and posterior DMN in SCD. Retrosplenial cortex mediated connectivity between hippocampus and DMN in controls but not SCD.	Decrease
Hayes et al.	2017	[42]	fMRI	Event-related visual memory encoding task	Voxelwise contrasts based on subsequently remembered items. Higher level analysis carried out with FMRIB’s Local Analysis of Mixed Effects.	Participants with and without SCD recruited from general population. However, participants with SCD saw a medical professional regarding their complaints prior to participation.	23 SCD, 41 control	Weaker task-related activity related to successful encoding in occipital, superior parietal, and cingulate cortex in SCD. No differences in retrieval performance.	Decrease
Hu et al.	2017	[43]	fMRI	Intertemporal decision task with an episodic imagination task embedded within	Voxelwise task contrasts for choice process and subjective valuation components of task. Group level factorial designs.	Participants with SCD recruited from memory clinic and reported decline in memory with onset in the past 5 years. Control participants recruited from the general population and reported no cognitive concerns.	20 SCD, 24 control	Poor temporal reward decision-making related to reduced hippocampal engagement in SCD. Lower insulae activation during task-switching in SCD.	Decrease
Mazzon et al.	2018	[44]	EEG	Eyes closed resting state and Rey’s Auditory Verbal Learning task	Kruskal-Wallis and Wilcoxon rank sum tests compared groups on regional relative band power.	Participants recruited from a memory center. Inclusion criteria for SCD included persistent memory complaints within the past 5 years, normal cognitive performance, and no psychiatric disease.	8 SCD, 7 control	Lower parietal β and γ band power in SCD during a memorization task.	Decrease
Yang et al.	2018	[45]	fMRI	Eyes closed resting state	Amplitude of low-frequency fluctuation evaluation. Support vector machine evaluation of ALFF for group discrimination.	Participants with SCD recruited from a memory clinic. Healthy controls recruited from general population. SCD determination based on the SCD international working group definition and made by experienced neurologists.	44 SCD, 57 control	Lower amplitude and fraction amplitude of low-frequency fluctuations in precuneus, anterior cingulum, and cerebellum in SCD.	Decrease
Lopez-Sanz et al.	2019	[46]	MEG	Eyes closed resting state	Source power spectra estimation and group classification based on source power analysis with regularized logistic regression with the Least Absolute Shrinkage and Selection Operator.	Participants recruited from a hospital neurology department, a center for the prevention of cognitive impairment, and a senior center. Cognitive concerns self-reported and SCD status determined by multidisciplinary panel.	91 SCD, 70 control	Lower relative α band power, predominantly in frontal regions, associated with SCD.	Decrease
Viviano et al.	2019	[47]	fMRI	Eyes closed resting state	Region-of-interest based functional connectivity analysis.	Participants with and without SCD recruited from general population. However, participants with SCD saw a medical professional regarding their complaints prior to participation.	35 SCD, 48 control	Lower functional connectivity between retrosplenial cortex and precuneus in SCD.	Decrease
Wang et al.	2019	[48]	fMRI	Eyes closed resting state	Vertex-based functional connectivity analysis and graph-theoretic approaches to the functional connectome.	Participants with SCD recruited from memory clinic and status determined by two psychiatrists according to the international working group standard proposed by Jessen et al. Participants without SCD recruited from general population.	32 SCD, 40 control	Lower subgraph centrality in occipital and paracentral regions in SCD.	Decrease
Babiloni et al.	2010	[49]	EEG	Eyes closed resting.	Spectral and cortical source analysis of EEG data.	Participants with SCD recruited from outpatient clinics with memory centers. Participants with SCD had *z*-scores > − 1.5 on cognitive battery. Controls also recruited from the clinics, but without and neurologic or psychiatric disease, and without social limitations.	53 SCD, 79 control	Greater frontal δ band amplitude, lower parietal and occipital θ and α1 (8–10.5 Hz) amplitude, and greater occipital α2 (10.5–13 Hz) amplitude in SCD.	Complex
Erk et al.	2011	[50]	fMRI	Visual memory encoding, recall, and recognition tasks. N-Back working memory task	Voxelwise task contrasts. Group comparison with ANOVA (full-factorial design). Family-wise error correction across whole brain at *p* < .05, or across a-prior hippocampal and dorsolateral prefrontal regions of interest.	Participants with SCD recruited from memory clinic and had informant corroboration. Healthy controls without complain recruited from the general population. All participants scored within 1.5 standard deviations on all subtests of the Consortium to Establish a Registry for Alzheimer’s Disease.	19 SCD, 20 control	Lower posterior hippocampal activation and greater right dorsolateral prefrontal cortex activation during recall in SCD. No differences in task performance or task-related activity during memory encoding. No group differences in N-back task.	Complex
Vega et al.	2016	[51]	fMRI	Resting state	Voxelwise seed-based functional connectivity. Second-level regression analysis associated cognitive complaint index with voxelwise connectivity.	Healthy, older, post-menopausal women recruited from general population. Participants identified as SCD if they endorsed > 20% of the items of the Cognitive Complaint Index and as control otherwise. Cognitive complaints evaluated as a continuous variable.	31 SCD (continuous)	Greater within executive control network and within middle temporal gyrus connectivity, and lower frontal cortex functional connectivity in SCD.	Complex
Lopez-Sanz et al.	2017	[52]	MEG	Eyes closed resting state	Source reconstruction and phase synchronization functional connectivity analysis.	Participants recruited from a hospital neurology department, a center for the prevention of cognitive impairment, and a senior center. Cognitive concerns self-reported and SCD status determined by multidisciplinary panel.	41 SCD, 39 control	Increased α-band connectivity between anterior regions and decreased α-band connectivity between posterior regions in SCD.	Complex
Jiang et al.	2018	[53]	fMRI	Eyes closed resting state	Vertex-based functional connectivity analysis.	Participants with SCD recruited from memory clinic and status determined by two psychiatrists according to the international working group standard proposed by Jessen et al., [1]. Participants without SCD recruited from general population.	42 SCD, 54 control	Lower vertex-wise index of functional criticality (a measure of signal standard deviation, within cluster correlation, and correlation with components outside a cluster) in the inferior temporal gyri and frontal poles, and greater criticality in precuneus, cingulate, parietal, and ventromedial prefrontal cortices in SCD.	Complex
Li et al.	2018	[54]	fMRI	Eyes open resting state	Whole-brain Pearson’s correlations and graph-theoretic methods to evaluate functional connectivity differences between groups.	Participants with SCD and healthy controls pulled from the Alzheimer’s Disease Neuroimaging Initiative (ADNI) database.	44 SCD, 40 controls	Participants with SCD exhibited greater degree centrality in hippocampus, and fusiform gyrus, and lower inferior parietal degree centrality. No differences between groups in eigen centrality. No differences between groups in amyloidopathy or tauopathy.	Complex
Xie et al.	2019	[55]	fMRI	Eyes closed resting state	Static and dynamic functional connectivity based on connectivity matrices derived from the Automated Anatomical Labeling atlas.	Participants with SCD recruited from a memory clinic. Healthy controls recruited from general population. SCD determination based on the SCD international working group definition and made by experienced neurologists.	40 SCD, 53 control	Centrality frequency (proportion of time a degree centrality hub region appeared in a dynamic functional connectivity analysis) differed between SCD and controls. Lower gyrus rectus and cingulum, and greater hippocampus, calcarine, lingual, and occipital centrality frequency in SCD.	Complex
Yan et al.	2019	[56]	fMRI	Eyes closed resting state	Multimodal (functional connectome and structural connectome via diffusion imaging) classifier training.	Participants with SCD recruited from a memory clinic. Healthy controls recruited from general population. SCD determination based on the SCD international working group definition and made by experienced neurologists.	39 SCD, 45 control	Classification accuracy for distinguishing SCD from controls ranged from 72 to 77% over a set of classifiers trained on connectome data. Important regions for classification included frontal, parietal, temporal, and hippocampal regions.	Complex
Dierks et al.	1997	[57]	EEG	Eyes closed resting.	EEG segmented into microstates based on the locations of centroids of electrical activity during spikes in spatial variance.	Participants with SCD recruited from memory clinic, did not exhibit difficulties in everyday living, and performed less than one standard deviation below age reference average on cognitive tasks. Control subjects did not harbor memory complaints and came from the general population.	31 SCD, 21 control	Duration of microstates, distinct spatial patterns of electrical activity, reduced in dementia. No difference between controls and SCD.	No difference
Lopez-Sanz et al.	2017	[58]	MEG	Eyes closed resting state	Source reconstruction and region-of-interest based functional connectivity analysis. Graph theoretic approaches utilized.	Participants recruited from a hospital neurology department, a center for the prevention of cognitive impairment, and a senior center. Cognitive concerns self-reported and SCD status determined by multidisciplinary panel.	55 SCD, 63 control	Decreased small-world characteristics of mild cognitive impairment brains compared to healthy controls in θ and β bands. Individuals with SCD exhibited a similar pattern, though not significantly different from controls.	No difference
Teipel et al.	2018	[59]	fMRI	Resting state	Pearson’s correlation based functional connectivity. Amplitude of low-frequency fluctuation evaluation. Evaluation of functional connectivity to distinguish SCD via cross-validated discrimination accuracy based on penalized logistic regression.	Participants recruited from the general population and grouped as SCD if they answered “Yes” to the questions “Are you complaining about your memory?” and “Is it a regular complaint that lasts more than 6 months?”	90 SCD, 80 controls	Functional connectivity did not reach significant discrimination accuracy for distinguishing SCD from controls.	No difference
Contreras et al.	2019	[60]	fMRI	Eyes closed resting state	Within- and between-network functional connectivity based on whole-brain parcellation.	Participants drawn from two cohorts. Participants with SCD had cognitive concerns but tested normally on cognitive tests. Controls had no significant cognitive concerns.	27 SCD, 31 control	No significant difference between controls and SCD.	No difference
Scarapicchia et al.	2019	[61]	fMRI	Eyes open resting state	Association between resting-state voxelwise BOLD variability and white matter hyperintensities older adults with and without SCD.	Participants drawn from ADNI database. SCD status determined by ADNI investigators. Participants self-reported cognitive concerns and scored > 15 on the first 12 items of the Cognitive Change Index. Control participants had no significant cognitive concerns.	19 SCD, 19 control	No significant difference between controls and SCD in blood-oxygen-level dependent variability. Higher white matter hyperintensity burden associated with greater variability in temporal regions for controls only.	No difference

As mentioned previously, SCD outcomes are varied, with some individuals reverting to a subjectively unimpaired state while others may develop dementia. Furthermore, there are additional factors that could influence perception and report of perceived cognitive decline, such as personality or depression [62–68]. However, for the analyses considered in this review, many research groups recruited samples from memory clinics, adhered to guidelines proposed by an international working group [1], and accounted for potential confounds either in recruitment or during data analysis. Therefore, we are confident that a synthesis of this literature reflects functional brain characteristics of SCD for individuals who may harbor incipient neurodegeneration and are at greater risk of developing dementia, and thus interpret the results within that assumption.

## Functional neuroimaging in subjective cognitive decline

### Task-related brain activity in subjective cognitive decline

Previous fMRI analyses that examined memory processing in older adults have observed lower hippocampal and greater dorsolateral prefrontal cortex task-related activity during memory retrieval, as well as greater task-related activity in dorsolateral prefrontal cortex and lower activity in superior parietal, cingulate, and occipital cortex during memory encoding in individuals with SCD [26, 42, 50]. These results suggest that SCD may arise from processing aberrations underlying either memory encoding or retrieval. As different analyses uncovered encoding or retrieval group differences, the ability to efficiently access stored information may diminish first for some while the ability to efficiently encode information may deteriorate earliest for others. This suggests that episodic memory encoding and retrieval processing alterations are independently sufficient for the subjective experience of decline, but neither are necessary.

In addition to episodic memory, fMRI analyses have also identified group differences in functional activity during working memory and other executive functions in SCD. Prior analyses suggest greater working memory-related activity in precuneus, middle frontal gyrus, and cingulate gyrus [29], and greater divided attention-related activity in thalamus, caudate, posterior cingulate, hippocampus, and parahippocampus [28] in individuals with SCD. Moreover, greater EEG alpha band power across electrodes, greater frontal theta power, and greater spatiotemporal wave activity during working memory have been observed in older adults with SCD, with greater alpha power and wave activity relating to lower verbal memory performance and reaction time, and greater reverse digit span performance [25]. Finally, greater MEG post-stimulus-related activity has been noted in older adults with SCD in parietal, temporal, occipital, motor, and dorsal prefrontal during a working memory task [27]. Overall, these analyses provide evidence that neural information processing disruptions in SCD do not just relate to memory but may also extend to executive functions.

Though many analyses have evaluated functional brain aberrations related to memory and executive functions, these processing disturbances may not be the sole drivers of SCD. Previously observed lower insula activation in individuals with SCD during a task that involved switching between imagination and temporal decision-making suggests decreased ability to control engagement of the default mode and executive control networks in SCD [43]. The default mode network is involved in episodic memory, theory of mind, and future planning [69], while the executive control network is involved in working memory, attention, and other executive functions [70, 71]. Lower insula activation during task switching [43], however, suggests that SCD may relate to inefficient salience network functioning, as the insulae are part of a network observed to send control signals to the default mode and executive control regions to modulate their engagement relative to internal and external task demands [72]. Thus, it is possible that the circuitry involved in memory and executive functions may operate efficiently in some individuals with SCD, but the ability to properly recruit different circuits at appropriate times diminishes.

Overall, tasked-based analyses suggest that cognitive processing alterations exist in individuals with SCD, but precise and accurate synthesis of the results remains difficult. Lower hippocampal task-related activity during retrieval [50] and decreased occipital and default mode network task-related activity during encoding [42] could either indicate local processing inefficiencies in the observed regions or indicate decreased network signaling to those regions during memory processing in SCD. This interpretation is based on multimodal imaging research that indicates that neuronal contributions to the blood-oxygen-level dependent (BOLD) response reflect metabolic demands of synaptic activity [73], which suggests that regional signal reflects local computation and processing of distal afferent input. This interpretation of the BOLD response also contextualizes observations of greater task-related activity in frontal regions during encoding or retrieval [26, 50], which could reflect either greater processing within regional microcircuits, or greater processing of afferent input to frontal regions. Greater processing of afferent input could reflect functional compensation [74]. Here, the neurocognitive Scaffolding Theory of Aging and Cognition would posit that cognitive ability remains relatively stable through aging despite neuronal declines, and the continuous recruitment of additional neuronal circuits, which may manifest as functional activity increases via neuroimaging analysis, may be responsible for stable cognitive ability through healthy aging and early disease states. Thus, functional activity increases in frontal regions in SCD could represent compensatory recruitment (more processing of afferent input) to aide processing deficiencies in circuits with neural insults. Greater executive task-related activity and greater alpha band power in individuals with SCD [25, 27–29] could also indicate explicit strategy use in response to self-knowledge of decline, rather than compensatory regional recruitment outside of cognitive control. Though, greater alpha band power in older adults with SCD under cognitive load could also reflect more automatic compensatory processing to nascent decline, an interpretation supported by increased band power relating to better executive performance [25].

However, another valid interpretation of greater regional task-related activity in SCD could be that computational failure in a circuit leads to a shifting of processing loads and computation to other regions. While the distinction between compensatory recruitment and shifting processing load is subtle, this reframing of interpretation would fit the Cascading Network Failure model of AD [20]. Furthermore, although compensatory processing is a common chorus across discussion sections, increased task-related activity could also reflect inefficient processing. Briefly, decreased network segregation or age-related functional dedifferentiation [75, 76], which could reflect less economical neural function in aging where maintenance of cognitive abilities may require greater BOLD signal [77, 78], could be accelerated in SCD and reflect inefficient information processing and noisy signal propagation across brain networks. Synaptic disruption, supported by observations of structural atrophy in SCD [79–86], could also result in unrefined noisy signal. Therefore, it could be that the increased task-related activity and alpha band power during executive functions reflects increased processing demands in individuals with SCD, possibly from inefficient signal processing and system noise, rather than compensatory mechanisms. For example, increased spatiotemporal wave activity [25] could indicate lower stability of distinct states of information processing, and greater alpha band power could indicate increased processing demands of attention without necessarily offering a compensatory advantage. Negative associations of alpha band power with verbal memory performance and reaction time [25] could support this interpretation.

Overall, the exact interpretations of complex patterns of increased and decreased task-related neural activity remain up for debate. However, existing theories and models of cognitively unimpaired aging and dementia development, e.g., the Scaffolding Theory of Aging and Cognition [74], the Dedifferentiation Theory of Aging [76], or the Cascading Network Failure Model of AD [20], offer valid interpretations for comparing the functional brains of older adults with and without SCD. There is also room for a general model that unifies concepts across these theories and models to explain functional alterations of the brain in SCD. Compensatory neural recruitment, non-compensatory network dedifferentiation, cascading network failures and processing load shifts, noisy signal propagation, and inefficient network signal processing could all occur in the same brain; one mechanism could influence observed neural activity in one region while a different mechanism affects the activity in another region.

### Brain network functional connectivity in subjective cognitive decline at wakeful rest

Variability and inconsistencies across the resting-state functional connectivity literature in SCD have resulted in a challenging picture to decipher. Some groups have reported greater connectivity, mainly within and between default mode network and medial temporal regions, in older adults with SCD [30, 31, 35, 36]. However, many groups have also observed lower connectivity between these regions in individuals with SCD [18, 76–79]. Others have reported no difference from control [60], or intricate patterns of greater and lower functional connectivity in individuals with SCD [51–55], suggesting that a complex reorganization of processing responsibilities across network nodes may occur. Multifaceted patterns of increased and decreased connectivity between regions could reflect breakdown in processing within a subnetwork that shifts processing load to more intact components of the broader network. Diffuse and frontally localized MEG alpha band power reductions have also been noted in older adults with SCD [39, 46], without group differences in the small world properties of theta and beta bands [58]. Moreover, lower subgraph centrality (a measure of weighted, closed walks starting and ending at a node, representing mid-scale connectivity) has been observed in occipital and paracentral regions in older adults with SCD [48].

Though the functional connectivity results appear incongruous, they do suggest that connectivity abnormalities occur in SCD that could represent information processing inefficiencies and could demarcate incipient dementia. The implications of elevated functional connectivity in SCD [30, 31, 35] are unclear but could reflect compensatory intrinsic signaling resulting from gray matter atrophy or other neural insult. This could reflect a neurocognitive scaffolding mechanism for maintaining stable internal mentation and memory functioning [74]. However, increased connectivity does not always confer greater cognitive performance and may reflect a shift in network properties that underlies poor memory performance [87]. Increased processing demands for internal mentation and memory at rest could arise due to noisier information traveling through memory systems [77]. Increasing regional metabolic demands from processing noisy information could serve as a mechanism that induces further neurodegenerative decline. Eventually, this neurodegenerative pressure could shift elevated functional connectivity early in SCD to decreased functional connectivity later in SCD.

Lower functional connectivity between default mode and medial temporal structures in SCD [18, 37, 38, 41, 47] could either reflect cohort differences compared to analyses noting greater functional connectivity in SCD [30, 31, 35, 36] or represent a later phase of SCD close to mild cognitive impairment conversion marked by decreased signaling capability between regions. While involved in episodic memory, cortical midline regions of the default mode network also mediate self-referential processing [69, 88]. Therefore, reduced connectivity could indicate either decreased computation related to self-awareness of memory processing or decreased memory processing directly. Furthermore, as cortical midline structures, hippocampus, and parahippocampus compose a memory system import for accessing episodic memories [89], and as decreased posterior hippocampal activation during memory recall has been noted in older adults with SCD [50], the aforementioned task and resting-state results together provide parallel evidence that issues in memory access processing are either a common or a sufficient component of SCD.

The confounding variables responsible for discrepancies in the results remain unclear; however, a few differences across samples could help explain the results. For example, [18] found lower fractional anisotropy in the superior longitudinal fasciculus and higher fractional anisotropy in the parahippocampal cingulum in individuals with SCD that significantly associated with the functional connectivity of cortical midline structures. Aberrant diffusion characteristics involving physical connections between memory and self-referential regions may distinguish this sample from the others and may reflect a mechanism for lower functional connectivity unrelated to mechanisms underlying increased connectivity [30, 31, 35]. Group differences in diffusion metrics do not explain the group differences in [47] though as there were no differences between the groups in neurite orientation dispersion and density [90] throughout the cingulum fiber tract. However, lower working memory performance for the SCD participants in that sample may have related to decreased connectivity.

Inconsistent results of increased and decreased functional connectivity may also reflect unmeasured confounds. The confounds are unlikely to relate to age as the average sample ages do not appear to drive the differences; they averaged between the mid-sixties to early seventies without a clear pattern of younger or older samples corresponding to particular group differences. Thus, the underlying reason for differences in cross-sectional results could reflect an important unmeasured variable. Though aforementioned sample differences in structural and cognitive measures may have accounted for some of the connectivity variability across studies, there is one commonly unreported variable which may capture a substantial portion of connectivity variance in SCD: time since the onset of perceived cognitive worsening.

**Fig. 1:**
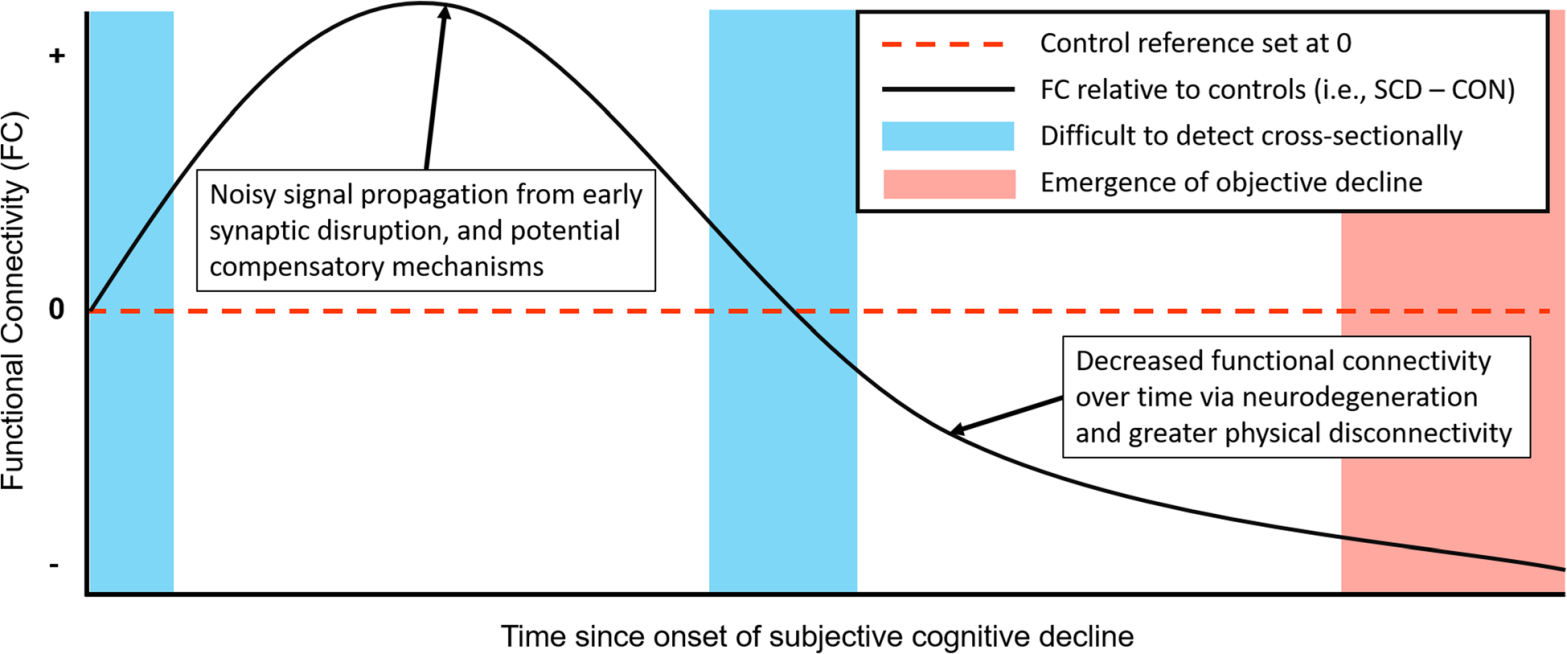
Possible trajectory of resting-state functional connectivity changes in subjective cognitive decline for default mode network and medial temporal regions based on cross-sectional analyses. Observations of both greater and lower functional connectivity in SCD could suggest a nonlinear change trajectory over the progression from subjective to objective impairment, highlighting the need for longitudinal analysis. Early synaptic disruption may lead to noisy signal propagation across systems or early compensatory processes that underlie elevated functional connectivity which may transition to lower functional connectivity as neurodegeneration progresses and objective decline become more apparent. Furthermore, this trajectory might shift depending on the brain regions evaluated as some groups report complex patterns of both higher and lower functional connectivity in SCD

Time since onset of SCD may be an important measure for both researchers and clinicians if brain change trajectories over the progression of SCD to objective decline are nonlinear. This suggests a possible reconciliation to the discrepancies of previous connectivity research in SCD. The results may not disprove each other, but rather, they may capture different stages of SCD; high or low network connectivity could reflect different phases of the underlying mechanisms responsible for the experience. Though speculative, and requiring longitudinal evaluation for verification, we propose that elevated network functional connectivity characterizes early SCD, which transitions to lower functional connectivity with time (see Fig. 1). Though, the time course of this nonlinear trajectory may emerge in some regions earlier than other, e.g., in regions most susceptible to early neurodegenerative change like the transentorhinal areas in AD [91]. This concept of elevated connectivity transitioning to lower connectivity is not novel, and reviews of dementia research have proposed this concept before [92]. However, here, we suggest that this trajectory reflects the underlying neurodegenerative process of the transition between cognitively unimpaired aging and dementia, and that SCD is the experience of this process.

Due to potential nonlinearities, the existence of inflection points in the trajectory of brain changes related to pathology could reduce differences between healthy older adults with and without SCD in cross-sectional analyses. This may not be the case for protein aggregates as they may not spontaneously clear. But for brain measures, such as functional integration, where either a positive or negative association with SCD could be meaningful, and where a change in the direction of association may be equally or more meaningful, the omission of variables related to length of time that participants have experienced SCD may pose a fundamental problem for data analyses. Because of possible nonlinearities in brain changes or other changes related to SCD, longitudinal analysis must drive the discussion regarding potential neurodegenerative-related brain changes in this population. Furthermore, ongoing longitudinal data collection efforts should retroactively gather years-since-onset information when possible, and new projects should collect this information from the start.

Although a nonlinear trajectory for functional connectivity change makes cross-sectional analysis difficult to interpret, there may be an unseen benefit to this potential change trajectory. Evaluation of an individual over multiple timepoints could help determine their progression though SCD, regardless of initial measurement values. For example, an individual at an early phase of SCD might exhibit increasing functional connectivity over time while an individual on the cusp of converting to mild cognitive impairment or dementia may exhibit decreasing connectivity over the time. This hypothetical trajectory requires confirmation, but nevertheless suggests that functional connectivity may be a valid method to track the progression of incipient neurodegenerative disease and may be useful regardless of the magnitude of the baseline measurement.

Finally, recruitment methods may also influence functional connectivity results. Perhaps individuals who are concerned about their cognitive ability and seek medical advice represent older adults with SCD more likely to harbor incipient neurodegeneration while individuals recruited from the general public may include SCD cases in an early disease phase, or cases due to neuroticism, poor sleep, or transient stressful life events. The extant literature suggests that recruitment methods may indeed contribute to SCD sample characteristics, with cohorts recruited from memory clinics exhibiting poorer neuropsychological performance, greater hippocampal atrophy, greater cortical atrophy over time, and greater depression than cohorts recruited from the general population that have not sought medical help [93–95]. However, as samples that have sought medical help have exhibited both increased and decreased functional connectivity cross-sectionally [18, 30, 47], the impact of the severity of cognitive complaint on functional metrics is not as clear. Future research should aim to further clarify if recruitment methodology is an important predictor of the type of functional connectivity pattern exhibited in SCD.

## Hypothetical functional model of SCD

**Fig. 2:**
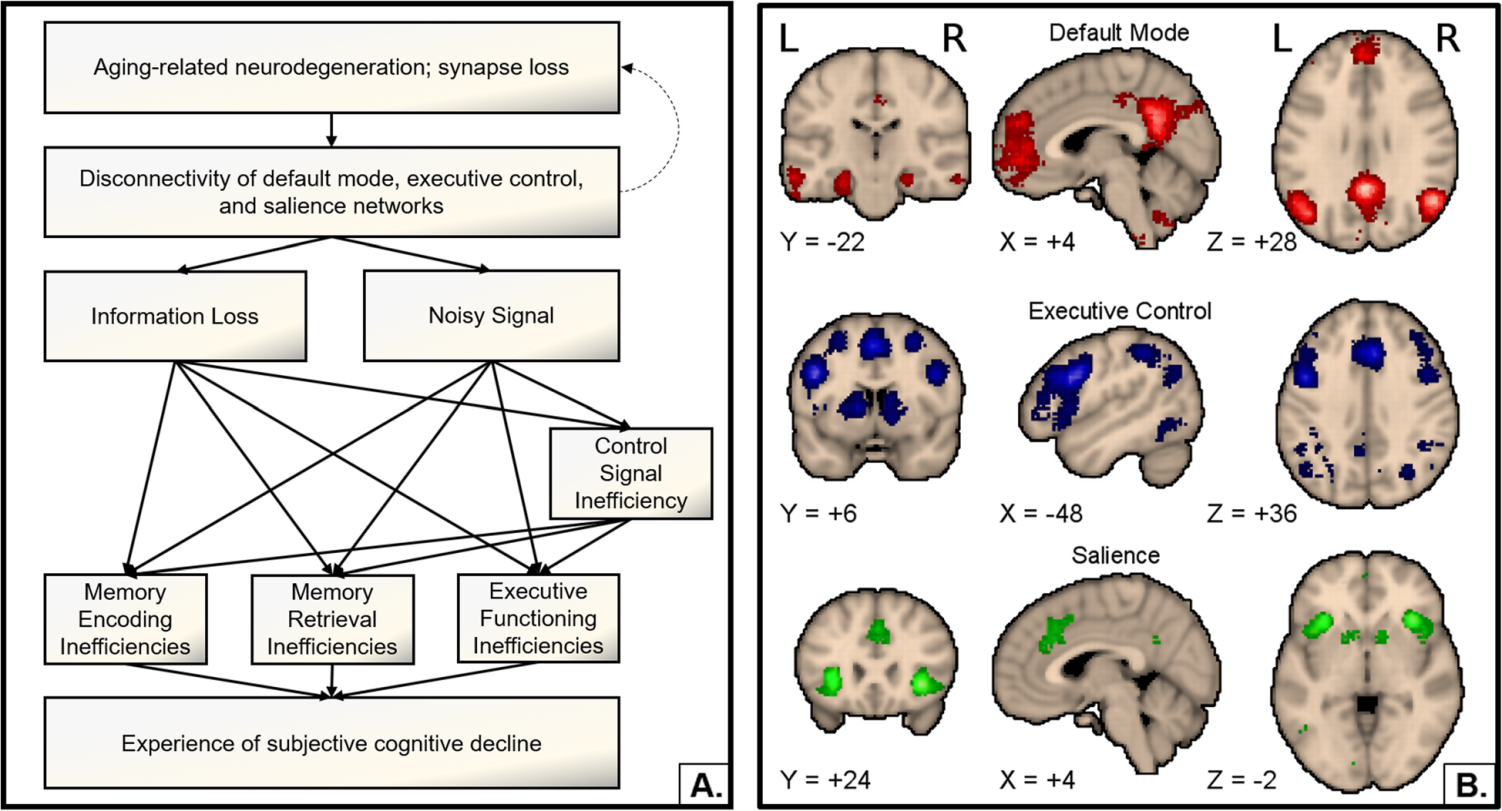
Proposed functional model of subjective cognitive decline. **a** Incipient neurodegeneration and synapse loss lead to disruption in short- and long-range functional connections within and between default mode, executive control, and salience networks. This disconnectivity may in turn lead to information loss and noisy signal propagation that directly disrupts memory and executive functions, and indirectly disrupts those functions though reduced ability of the salience network to modulate the activity of the default mode and executive control networks with control signals. Disrupted memory and executive functioning in turn results in the personal experience of deteriorating cognitive ability. **b** Default mode (red), executive control (blue), and salience (green) networks visualized with Neurosynth uniformity test images [110]. Search terms included “default network,” “executive network,” and “salience network.” *Z*-score thresholds set at 6

As individuals with SCD may revert to a subjectively normal state, maintain cognitive performance over time, or develop dementia, models of SCD that reflect functional connectivity change related to progressive neurodegeneration implicitly refer to the latter group. While SCD outcomes are heterogenous, we argue that functional neuroimaging has promise as a marker for incipient neurodegeneration and for progression of cognitive decline, distinguishing between those that develop dementia and those that do not. Therefore, we propose the following model to account for common features of SCD based on the aforementioned functional neuroimaging results (Fig. 2).

The first node of the model represents age-related neurodegeneration; at the cellular level, synapse loss could occur before appreciable cell death. Thus, in early SCD, this node may not represent cell loss, but rather physical disconnectivity. This disconnectivity could impact short-range connection within a region or could impact long-range connections that mediate the signaling between different whole-brain networks. For SCD, we speculate that disruptions occur in the hippocampus and surrounding medial temporal lobe through observations of altered task-related hippocampal activity and resting connectivity [38, 43, 50] and, although not the focus of this review, observations of structural atrophy [79–86]. As hippocampal atrophy is a common feature of AD, this may help identify adults with SCD that could develop AD specifically while other neurodegenerative patterns could indicate dementia development due to a different cause. We also speculate that long-range connectivity alterations occur between regions of the default mode, executive control, and salience networks based on diffuse task-related and resting functional connectivity alterations across studies [18, 26, 30, 31, 35, 38, 41–43, 47, 50].

Altered connectivity, both at the local and global level, presumably carries two major consequences: (1) information loss from signal not reaching downstream targets in a global circuit, and (2) noisy signals that reach downstream targets, but arise due to inadequate processing and refinement from local circuits and whole-brain networks. Information loss and noisy signal could lead to processing inefficiencies in control signals from the salience network that mediate memory and executive processes, as well as direct processing inefficiencies in memory encoding or retrieval, and executive functioning. Specific processing inefficiencies may be highly individualized, and the ordering of component failure or regional decline may differ between individuals, but all processing inefficiencies may be sufficient for the experience of SCD, and subtle objective decline that neuropsychological tests may not be sensitive enough to detect. Functional neuroimaging allows for the visualization of these inefficiencies through observation of network functional connectivity and task-related neural activity aberrations, and continued cross-sectional and longitudinal evaluation of network connectivity and activity can help refine this model.

While we place loss of synaptic connections as the first node in the model and speculate that they could have a causal role in disrupting network signaling, it is reasonable to order the events in reverse. Disrupted signaling between brain regions may affect trophic signaling between pre- and post-synaptic neurons and downstream processes related to synaptic maintenance [96, 97], thus having a causal role in neuropil loss. Hebb’s postulate stated colloquially: “Cells that fire together, wire together” [98]*.* Therefore, temporal ordering of events requires exploration, as does the possibility that a positive feedback loop exists where synaptic disconnectivity and gray matter atrophy through neuropil and cell loss amplify functional signaling disruption between regions, which in turn exacerbates cell and neuropil loss by disrupting synaptic maintenance processes.

While we emphasize that the proposed and alternative functional models of SCD are non-specific to neurodegenerative etiology and outcome, AD may be the dominant dementia etiology [19]; thus, it will be important to extend this model, or create a parallel model, to account for individuals with SCD that specifically exhibit AD pathology. As network failure may occur before amyloid-β accumulation [20], SCD has variable association with amyloidopathy and tauopathy [11–18], and detection of aberrant amyloid-β and tau may precede observable structural decline [99], we speculate that our model could fit in-between the Cascading Network Failure [20] and Pathological Cascade [99] models of AD for older adults with SCD that develop AD specifically. Here, network failure and complex reorganization of processing loads across whole-brain networks could influence metabolic and molecular processes related to AD pathology and neurodegeneration while simultaneously accounting for the experience of decline. Analyses reporting functional connectivity differences between older adults with and without SCD without finding group differences in amyloid or tau deposition [18, 54] suggest that functional brain alterations and perception of cognitive decline could co-occur before AD pathology detection. Finally, this model could be extended to account for disease-specific outcomes in general; however, future research must determine the patterns of functional connectivity change that are sensitive enough to predict specific outcomes before this model is extended.

## Future directions

While cross-sectional analyses have evaluated default mode and medial temporal functional connectivity, evaluation of other brain networks that SCD may impact remains sparse. Though the medial temporal lobes and default mode network may be sensitive to distinguish SCD from unimpaired, healthy aging, some individuals may display connectivity aberrations in other networks, such as the salience or executive control networks, before default mode network disruption occurs. For example, it is possible that the location of the earliest synaptic disruptions is determined by the specific cognitive domain of the initial complaints. Subjective decline in executive and attentional processing may relate to executive and salience network synaptic disruption prior to memory system disruption. The extant functional neuroimaging literature on SCD largely focuses on memory complaints and does not generally address other cognitive domains perceived as affected. Nevertheless, previous research suggests that connectivity disruption within the salience network, and between the salience network and other networks, may occur in older adults with SCD [43]. Salience network gray matter atrophy occurs at a greater rate in cognitively unimpaired aging than the rest of the brain, and atrophy is even greater in mild cognitive impairment and dementia, and this atrophy may occur alongside lower functional connectivity between salience and executive control network regions and greater connectivity between salience and default mode network regions [100]. If SCD indicates incipient dementia, greater salience network atrophy and connectivity alterations could occur compared to healthy aging and serve as a prognostic marker for further dementia development. In addition, subtle differences in cognitive performance between adults with and without perceived cognitive decline exist [101], and salience network disruption may help explain these group differences, possibly through failure to effectively engage the executive control networks for cognitive tasks while disengaging the default mode network. As mentioned previously, [43] found lower insula activation in individuals with SCD during a task that involved switching between task demands handled by default mode regions and task demands handled by executive control regions. Thus, there is evidence to support exploring connectivity between the default mode, salience, and executive control networks in SCD.

Furthermore, as mentioned earlier, longitudinal analyses must drive the discussion of functional connectivity change trajectories as some groups have observed greater functional connectivity in older adults with SCD compared to controls while other groups have observed lower functional connectivity. These results suggest a possible nonlinear change in brain network functional integration across the course of SCD. However, the only way to verify that speculation would be extensive longitudinal analysis.

While we emphasize the importance of longitudinal analyses to understand trajectories of functional connectivity change, there are important cross-sectional analyses that could use existing data to help clarify patterns of functional connectivity in SCD. However, they would require coordination across research groups. Currently, a meta-analysis of the functional neuroimaging literature in SCD is infeasible due to too many methodological inconsistencies (e.g., different network definitions, preprocessing pipelines, type of functional connectivity metric utilized, specific regions reported or evaluated). However, if research groups interested in SCD coordinated a common preprocessing and analysis pipeline to evaluate connectivity within and between whole-brain networks, the results could be combined in a pseudo-meta-analytic framework.

In addition to underexplored network connectivity in SCD, the full extent of functional differences during cognitive tasks between older adults with and without perceived decline remains unknown, and the field has yet to report on many detailed components of memory and other cognitive domains through functional neuroimaging. For example, although the extant literature to date has explored memory encoding and retrieval through visual and verbal means, these tasks have lacked explicit spatial components, and some spatial memory processing occurs in the medial temporal lobes [102]. Evaluation of spatial memory encoding and retrieval may be sensitive to detect hippocampal and medial temporal alterations in SCD that may serve as markers of further decline. Furthermore, continued evaluation of memory encoding and retrieval, working memory, and attention is necessary to understand the neural underpinnings of SCD.

Finally, although current neuroimaging resolutions only allow for macro-level evaluation of whole-brain network integration, it is still important to speculate on circuit-level changes that may occur in SCD as information processing ultimately occurs at the synaptic level. The circuitry of the medial temporal lobes may be important for understanding SCD for a multitude of reasons, including previously mentioned connectivity aberrations [30, 35, 38, 47], but also because T1-weighted and diffusion-weighted imaging analyses indicate that neuropil density and gray matter volume may be lower in entorhinal, perirhinal, parahippocampal, and hippocampal regions compared to controls [79–86, 103, 104]. Aberrant functional connectivity and significant atrophy within hippocampal circuitry may emerge as appropriate prognostic signals for disease, and the first subfield to critically evaluate may be cornu ammonis 1 as many reports suggest inward deformation of the subfield in older adults with SCD [105–107]. Unfortunately, typical functional connectivity analyses do not have sufficient spatial resolution to determine which hippocampal subfields or which hippocampal and entorhinal cell layers exhibit the greatest differences in connectivity between adults with and without SCD. Even with 2-mm isotropic voxels, subject movement, partial voluming, and spatial smoothing during image processing could smear signal from different hippocampal subregions together, in addition to smearing entorhinal and hippocampal signal. Thus, it is difficult to resolve whether disrupted communication between hippocampus and distant cortex is a downstream result of computation disruption within the hippocampus proper, disruption between direct hippocampus to cortex connections, or disrupted connectivity between entorhinal and parahippocampal cortex and other regions. Nevertheless, high-resolution, submillimeter fMRI is undergoing active development and analyses are uncovering functional organization across columnar and laminar cortical structures [108, 109]; this method could resolve aforementioned issues and evaluate hippocampal subfield connectivity. Combination of structural and functional methods could uncover mechanisms underlying SCD. For example, if diffeomorphic mapping finds inward deformation of cornu ammonis 1 in person with SCD that corresponds to decreased connectivity between cornu ammonis 1 and entorhinal cortex through submillimeter imaging, but intact communication between hippocampal subfields, then the results would indicate that hippocampal processing is largely intact but input to—and output from the hippocampus—may be aberrant in incipient neurodegenerative disease. Conversely, intact communication between entorhinal cortex and hippocampal subfields, but aberrant communication between subfields, would indicate that hippocampal processing disruption, rather than input or output disruption, initially occurs. Though speculative, functional neuroimaging in the future may allow for understanding connectivity disruption at the circuit level.

## Conclusion

SCD is a risk factor for dementia marked by perceived worsening of cognitive ability without observable deficit. The extant task-related activation and resting-state functional connectivity literature suggest that SCD relates to functional alterations in brain regions important for memory, such as retrosplenial cortex and hippocampus. Though, subtle disruption in memory encoding, retrieval, or other cognitive processes may all be sufficient for the experience of cognitive decline. Furthermore, aberrant task-related activation during memory and executive functioning tasks and aberrant functional connectivity in older adults with SCD suggest that information processing alterations may occur in the salience, executive control, and default mode networks. Finally, more research must occur on the functional characteristics of the brain in SCD to understand prognostic markers for dementia, and longitudinal evaluation of functional brain characteristics in SCD must occur specifically to determine trajectories of change that could serve as prognostic and diagnostic markers of objective decline.

## Data Availability

Not applicable.

## Abbreviations

AD: Alzheimer’s disease
ADNI: Alzheimer’s Disease Neuroimaging Initiative
BOLD: Blood-oxygen-level dependent
CON: Control
EEG: Electroencephalography
fMRI: Functional magnetic resonance imaging
MEG: Magnetoencephalography
SCD: Subjective cognitive decline
